# Association between the serum uric acid-to-high-density lipoprotein cholesterol ratio and gynecological cancer history: A cross-sectional study of NHANES 2003–2016

**DOI:** 10.1097/MD.0000000000050250

**Published:** 2026-08-21

**Authors:** Zelong Li, Hui Xu, Sensen Shi

**Affiliations:** a Department of Gynecologic Oncology, Fujian Maternity and Child Health Hospital, College of Clinical Medicine for Obstetrics & Gynecology and Pediatrics, Fujian Medical University, Fuzhou, Fujian, China; b Department of Oncology, The 900th Hospital of the Joint Logistic Support Force, PLA, Fuzhou, China.

**Keywords:** cancer history, cross-sectional study, gynecological cancer, NHANES, uric acid-to-HDL-C ratio

## Abstract

Serum uric acid (sUA) and high-density lipoprotein cholesterol (HDL-C) reflect complementary aspects of metabolic and inflammatory status. The uric acid-to-HDL-C ratio (UHR) has been investigated in cardiometabolic disorders, but evidence regarding gynecological cancer remains limited. We examined the association between UHR and a self-reported history of cervical, ovarian, or uterine cancer among U.S. women. We analyzed cross-sectional data from 7 NHANES cycles (2003–2004 through 2015–2016). The complete-case sample comprised 13,775 women, including 369 who reported a history of cervical, ovarian, or uterine cancer. UHR was calculated as [sUA (mg/dL)/HDL-C (mg/dL)] × 100. Survey-weighted logistic regression was used to estimate prevalence odds ratios (ORs) for UHR modeled continuously and by quartiles. Model 2 adjusted for age and race/ethnicity, and model 3 additionally adjusted for educational attainment, poverty–income ratio, alcohol use, smoking status, body mass index, oral contraceptive use, pregnancy history, and marital status. Restricted cubic spline (RCS), exploratory subgroup, receiver operating characteristic (ROC), and *E*-value sensitivity analyses were also performed. Women with a self-reported history of gynecological cancer had descriptively higher UHR values than those without such a history. In the fully adjusted model, each 1-percentage-point increase in UHR was associated with 3% higher prevalence odds (OR 1.03, 95% CI: 1.00–1.05; *P* = .03). However, none of the estimates for Q2–Q4 differed significantly from that for Q1 in model 3, although the *P* value for trend was < .05. RCS analysis provided no evidence of nonlinearity. The AUCs were 0.560 for UHR, 0.538 for sUA, and 0.554 for HDL-C, indicating poor discrimination. The *E*-value was 1.20 for the point estimate and 1.00 for the confidence-limit closest to the null, indicating limited robustness to unmeasured confounding. Higher UHR was weakly associated with greater prevalence odds of a self-reported history of gynecological cancer. The small effect size, nonsignificant fully adjusted quartile estimates, poor discriminatory performance, and susceptibility to residual confounding do not support the use of UHR for screening, early detection, or individual risk prediction. Prospective studies with validated outcomes and more comprehensive control of confounding are warranted.

## 1. Introduction

Uric acid is the final product of purine metabolism in humans. Although circulating urate can have antioxidant properties, higher concentrations are also associated with oxidative stress, inflammation, and cardiometabolic or renal dysfunction.^[1]^ Cross-sectional NHANES studies have additionally reported associations between higher sUA and female infertility, but such findings do not establish temporality or causality.^[2]^

High-density lipoprotein cholesterol (HDL-C) is a routinely measured lipid marker. HDL particles participate in reverse cholesterol transport and have context-dependent antioxidant and anti-inflammatory functions.^[3]^ Lipoprotein metabolism is altered in many cancers, and lower HDL-C has been associated with cancer occurrence and prognosis in observational studies; however, these associations may reflect confounding, reverse causation, or disease-related metabolic change.^[4,5]^

Globally, cervical, corpus uteri, and ovarian cancers contribute substantially to the cancer burden among women.^[6]^ Their etiologies differ, but metabolic factors – particularly obesity and metabolic syndrome – have been associated most consistently with corpus uteri/endometrial cancer.^[7]^ UHR combines sUA and HDL-C and has been studied as a metabolic marker in diabetes and metabolic syndrome.^[8]^ Although inflammation and oxidative stress provide biological plausibility for an association, existing evidence does not establish a specific UHR threshold that activates NF-κB or directly promotes gynecological tumor proliferation.

We therefore examined the cross-sectional association between UHR and self-reported history of cervical, ovarian, or uterine cancer in NHANES 2003–2016. The objectives were to estimate adjusted prevalence ORs, explore the functional form of the association, and describe – rather than validate – the discrimination of UHR compared with its components. The study was not designed to evaluate incidence, progression, early detection, or clinical prediction.

## 2. Materials and methods

### 2.1. Data source and study population

**Figure 1.**
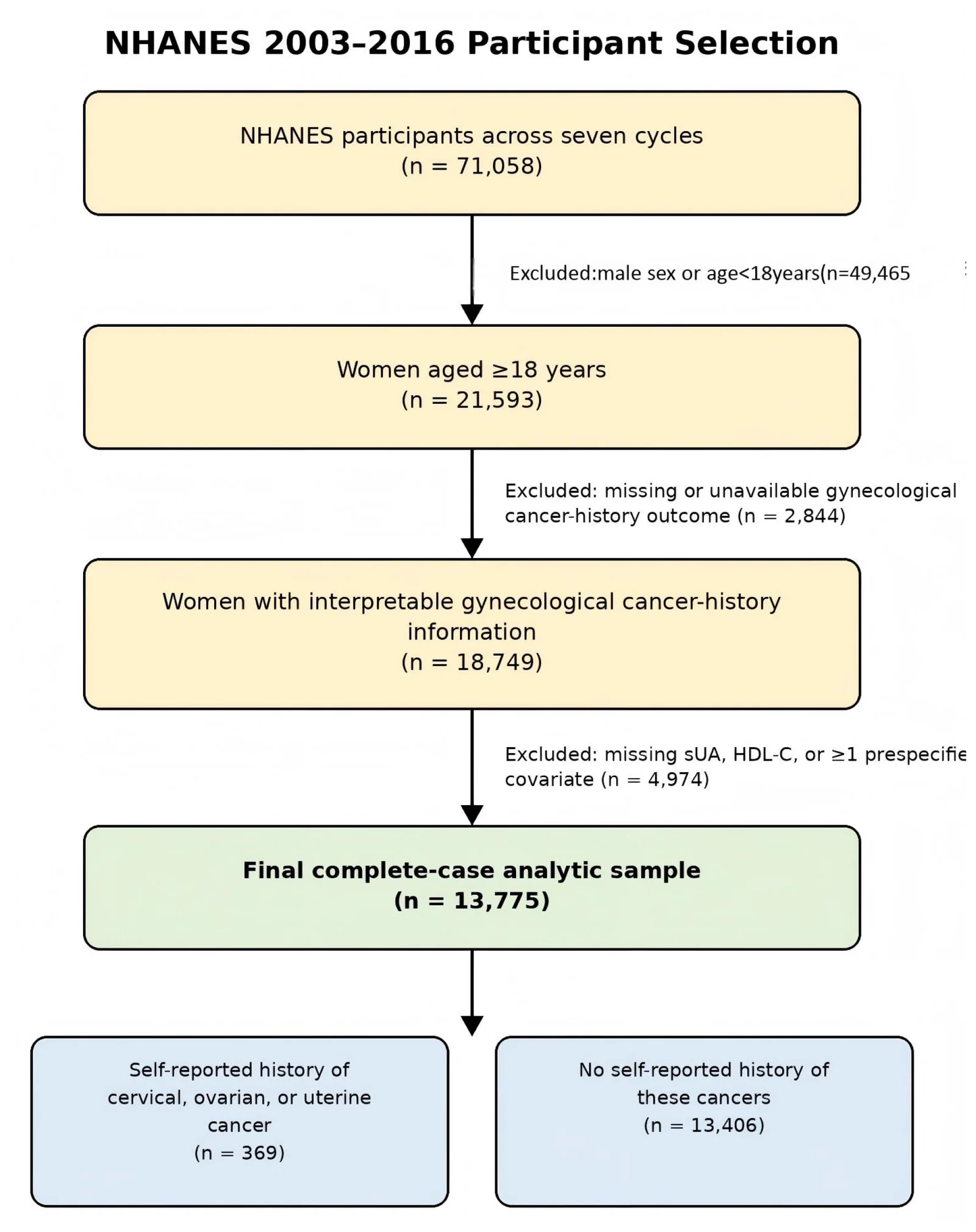
Flowchart of NHANES participant selection. The intermediate group (n = 18,749) represents women with interpretable information on gynecological cancer history, not women with gynecological cancer. NHANES = National Health and Nutrition Examination Survey.

This study used publicly available data from the National Health and Nutrition Examination Survey (NHANES), a biennial program conducted by the National Center for Health Statistics (NCHS) of the Centers for Disease Control and Prevention (CDC). NHANES uses a stratified, multistage probability sampling design to obtain a nationally representative sample of the noninstitutionalized US population. We combined data from 7 survey cycles conducted between 2003–2004 and 2015–2016, comprising an initial sample of 71,058 participants. We excluded males, participants younger than 18 years, and participants with missing data on serum uric acid, HDL-C, gynecological cancer history, or relevant covariates. Women who reported a physician diagnosis of cervical, ovarian, or uterine cancer were classified as having a gynecological cancer history; all other eligible women were classified as not having such a history. The final complete-case sample included 13,775 women, of whom 369 reported a gynecological cancer history and 13,406 did not. The participant-selection process is shown in Figure 1. NHANES protocols were approved by the NCHS Research Ethics Review Board, and all participants provided written informed consent. Because the datasets are publicly available and de-identified, no additional institutional review board approval was required for this secondary analysis.

The NHANES datasets are publicly available from the NCHS website (https://www.cdc.gov/nchs/nhanes/).

### 2.2. Exposure and outcome definitions

Serum uric acid was measured using a timed-endpoint method on the LX20 analyzer. Uric acid was oxidized by uricase to generate hydrogen peroxide, which subsequently formed a colored product through a peroxidase-catalyzed reaction; absorbance was measured at 520 nm. HDL-C was measured enzymatically using polyethylene glycol-modified cholesterol oxidase, with the resulting colored product quantified photometrically at 600 nm. Detailed laboratory quality-assurance and quality-control procedures are available on the NHANES website. UHR was calculated as [sUA (mg/dL)/HDL-C (mg/dL)] × 100 and expressed as a percentage. Sex was determined during the household interview. Gynecological cancer history was ascertained from responses to 2 Medical Conditions Questionnaire items: “Have you ever been told by a doctor or other health professional that you had cancer or a malignancy of any kind?” and “What kind of cancer was it?” Participants reporting cervical, ovarian, or uterine cancer were classified as having the outcome of interest.

### 2.3. Covariates

Covariates were selected a priori from variables that were available and harmonizable across all 7 survey cycles: age, race/ethnicity, educational attainment, poverty–income ratio (PIR), alcohol use, smoking status, body mass index (BMI), ever use of oral contraceptive pills, pregnancy history, and marital status. Age was modeled as a continuous variable in regression analyses and categorized only for descriptive and exploratory subgroup analyses. BMI was categorized as <18.5, 18.5 to 24.9, and ≥25.0 kg/m^2^, and PIR as <1, 1 to 3, and >3. Menopausal status, hormone therapy, family history, HPV exposure or vaccination, diabetes, renal function, and inflammatory markers were not included in the original analytic dataset; their omission may have resulted in residual confounding, as discussed in the limitations of this study.

### 2.4. Statistical analysis

All analyses accounted for the NHANES sampling weights, strata (SDMVSTRA), and primary sampling units (SDMVPSU). For the 7 combined cycles, the 2-year mobile examination center weight was divided by 7 in accordance with NHANES guidance. Descriptive statistics are presented as survey-weighted means with standard errors, medians with interquartile ranges, or weighted proportions, as appropriate. Between-group comparisons were performed using survey-weighted Wald tests for continuous variables and Rao–Scott chi-square tests for categorical variables. Estimates are reported with 95% confidence intervals.

Survey-weighted logistic regression was used to estimate prevalence ORs for the association between UHR and gynecological cancer history. Model 1 was unadjusted; model 2 adjusted for age and race/ethnicity; and model 3 additionally adjusted for educational attainment, PIR, alcohol use, smoking status, BMI, oral contraceptive use, pregnancy history, and marital status. UHR was modeled both continuously, per 1-percentage-point increase, and by quartiles. Tests for trend assigned each quartile its survey-weighted median UHR value and modeled this value continuously. RCS models were used to assess departure from linearity. Given the 369 outcome events, subgroup analyses were restricted to prespecified strata defined by age, BMI, smoking status, and oral contraceptive use. Effect modification was assessed using interaction terms rather than within-stratum *P* values. Because no correction for multiple testing was applied, subgroup findings were considered exploratory.

ROC analyses compared the apparent, unvalidated discrimination of UHR, sUA, and HDL-C for prevalent self-reported gynecological cancer history. AUC (area under the curve) values close to 0.5 were interpreted as indicating poor discrimination rather than evidence of clinical predictive utility. As a sensitivity analysis for unmeasured confounding, *E*-values were calculated for the fully adjusted continuous-UHR OR and for the confidence limit closest to the null.^[9]^ Analyses were conducted using R-based survey procedures within DecisionLinnc version 1.1.6.7. All tests were 2-sided. *P* < .05 was interpreted in conjunction with the magnitude of the effect, the width of the confidence interval, and the potential for multiplicity.

## 3. Results

### 3.1. Baseline characteristics

The complete-case sample included 13,775 women, of whom 369 (2.7% unweighted) reported a history of cervical, ovarian, or uterine cancer. Women in the cancer-history group were older and differed from those in the comparison group with respect to several sociodemographic and behavioral characteristics. sUA, HDL-C, and UHR also differed descriptively between groups; the reported mean UHR was 9.09% in the cancer-history group and 8.28% in the comparison group. These unadjusted differences should not be interpreted as evidence of an etiological relationship. Weighted and unweighted characteristics are presented in Tables 1 and 2.

**Table 1:** Basic characteristics between gynecological tumor and non-gynecological tumor groups enrolled in the NHANES cross-sectional study.

Characteristic	Total (n = 13,775)	Non-gynecological tumor (n = 13,406)	Gynecological tumor (n = 369)	*P* value
Age (%)				
18–45	6668 (48.41)	6536 (48.75)	132 (35.77)	**<.001**
46–60	3300 (23.96)	3188 (23.78)	112 (30.35)	
>60	3807 (27.64)	3682 (27.47)	125 (33.88)	
Race (%)				
Mexican American	2364 (17.16)	2311 (17.24)	53 (14.36)	**<.001**
Non Hispanic Black	2894 (21.01)	2852 (21.27)	42 (11.38)	
Non Hispanic White	6074 (44.09)	5847 (43.61)	227 (61.52)	
Other Hispanic	1269 (9.21)	1237 (9.23)	32 (8.67)	
Other race	1174 (8.52)	1159 (8.65)	15 (4.07)	
Education attainment (%)				
Less than high school graduate	3353 (24.34)	3239 (24.16)	114 (30.89)	
High school graduate	3039 (22.06)	2946 (21.98)	93 (25.20)	
College above	7383 (53.60)	7221 (53.86)	162 (43.90)	**.001**
Ratio of income to poverty level(%)				
<1	3130 (22.72)	3023 (22.55)	107 (29.00)	**.009**
1–3	5786 (42.00)	5636 (42.04)	150 (40.65)	
>3	4859 (35.27)	4747 (35.41)	112 (30.35)	
Alcohol group (%)				
No	5460 (39.64)	5307 (39.59)	153 (41.46)	.501
Yes	8315 (60.36)	8099 (60.41)	216 (58.54)	
Smoking group (%)				
No	8671 (62.95)	8512 (63.49)	159 (43.09)	**<.001**
Yes	5104 (37.05)	4894 (36.51)	210 (56.91)	
BMI group (%)				
<18.5	256 (1.86)	245 (1.83)	11 (2.98)	**.037**
18.5–24.9	3887 (28.22)	3801 (28.35)	86 (23.31)	
25	9632 (69.92)	9360 (69.82)	272 (73.71)	
Taken birth control pills (%)				
No	4451 (32.31)	4344 (32.40)	107 (29.00)	.186
Yes	9324 (67.69)	9062 (67.60)	262 (71.00)	
Pregnant status (%)				
No	2130 (15.46)	2102 (15.68)	28 (7.59)	**<.001**
Yes	11,645 (84.54)	11,304 (84.32)	341 (92.41)	
Marital status (%)				
Married/living with partner	7611 (55.25)	7416 (55.32)	195 (52.85)	.374
Living alone	6164 (44.75)	5990 (44.68)	174 (47.15)	
Serum uric acid (mg/dL)	4.7 (3.9–5.5)	4.6 (3.9–5.5)	4.8 (4.1–5.7)	**.008**
HDL (mg/dL)	56 (46–67)	56 (46–68)	53 (44–63)	**<.001**
UHR (%)	8.30 (6.28–11.06)	8.28 (6.27–11.03)	9.09 (6.77–12.26)	**<.001**

Bold values indicate statistical significance (*P* < .05).

BMI = body mass index, HDL = high-density lipoprotein, UHR = uric acid-to-high-density lipoprotein cholesterol ratio, NHANES = National Health and Nutrition Examination Survey.

**Table 2:** Basic characteristics between gynecological tumor and non-gynecological tumor groups, weighted.

Characteristic	Total** N = 8,30,59,382	Non-gynecological tumor** N = 8,07,25,411	Gynecological tumor** N = 23,33,971	*P*-value**
Serum uric acid (mg/dL)	4.60 (3.90–5.50)	4.60 (3.90–5.50)	4.70 (4.10–5.70)	**.032**
UHR (%)	8.10 (6.17–10.82)	8.08 (6.15–10.80)	9.04 (6.56–11.62)	**.005**
HDL (mg/dL)	57.00 (47.00–68.00)	57.00 (47.00–68.00)	54.00 (45.00–65.00)	**.017**
Age (%)				**<.001**
>60	1,81,13,584 (21.81)	1,74,49,252 (21.62)	6,64,331 (28.46)	
18–45	4,18,99,621 (50.45)	4,10,47,449 (50.85)	8,52,172 (36.51)	
46–60	2,30,46,178 (27.75)	2,22,28,710 (27.54)	8,17,468 (35.02)	
Race (%)				**.002**
Mexican American	64,61,047 (7.78)	63,29,680 (7.84)	1,31,366 (5.63)	
Non Hispanic Black	97,25,399 (11.71)	95,95,080 (11.89)	1,30,318 (5.58)	
Non Hispanic White	5,72,16,290 (68.89)	5,53,46,602 (68.56)	18,69,688 (80.11)	
Other Hispanic	42,26,507 (5.09)	41,33,810 (5.12)	92,697 (3.97)	
Other race	54,30,140 (6.54)	53,20,239 (6.59)	1,09,901 (4.71)	
Education attainment (%)				**.043**
College above	5,17,00,651 (62.25)	5,04,05,760 (62.44)	12,94,891 (55.48)	
High school graduate	1,82,84,795 (22.01)	1,77,22,627 (21.95)	5,62,168 (24.09)	
Less than High school graduate	1,30,73,937 (15.74)	1,25,97,024 (15.60)	4,76,913 (20.43)	
Ratio of income to poverty level (%)				**.019**
<1	1,27,50,376 (15.35)	1,22,28,674 (15.15)	5,21,702 (22.35)	
1–3	3,08,46,406 (37.14)	3,00,10,340 (37.18)	8,36,066 (35.82)	
>3	3,94,62,601 (47.51)	3,84,86,398 (47.68)	9,76,203 (41.83)	
Alcohol group (%)				.307
No	2,64,39,954 (31.83)	2,56,31,260 (31.75)	8,08,694 (34.65)	
Yes	5,66,19,429 (68.17)	5,50,94,152 (68.25)	15,25,277 (65.35)	
Smoking group (%)				**<.001**
No	4,98,17,650 (59.98)	4,89,73,522 (60.67)	8,44,128 (36.17)	
Yes	3,32,41,733 (40.02)	3,17,51,889 (39.33)	14,89,843 (63.83)	
BMI group (%)				.181
<18.5	16,95,982 (2.04)	16,10,826 (2.00)	85,156 (3.65)	
18.5–24.9	5,48,93,765 (66.09)	5,33,09,160 (66.04)	15,84,605 (67.89)	
25	2,64,69,635 (31.87)	2,58,05,425 (31.97)	6,64,210 (28.46)	
Taken birth control pills (%)				.219
No	2,12,47,675 (25.58)	2,07,29,191 (25.68)	5,18,483 (22.21)	
Yes	6,18,11,708 (74.42)	5,99,96,220 (74.32)	18,15,488 (77.79)	
Pregnant status (%)				**.003**
No	1,54,14,425 (18.56)	1,51,79,653 (18.80)	2,34,772 (10.06)	
Yes	6,76,44,957 (81.44)	6,55,45,759 (81.20)	20,99,198 (89.94)	
Marital status (%)				.620
Married/living with partner	5,05,64,640 (60.88)	4,91,74,273 (60.92)	13,90,367 (59.57)	
Living alone	3,24,94,742 (39.12)	3,15,51,138 (39.08)	9,43,604 (40.43)	

Data are expressed as median (IQR) for continuous variables and percentage (%) for categorical variables.

Continuous variables with non-normal distribution were compared between groups using the Wilcoxon rank-sum test. Categorical variables were expressed as frequencies and percentages and compared using the Pearson x^2^ test.

Gynecological tumor including: Ovarian cancer, Endometrial cancer, Cervical cancer.

Bold values indicate *P* < .05.

BMI = body mass index, HDL = high-density lipoprotein, IQR = interquartile range, UHR = uric acid-to-high-density lipoprotein cholesterol ratio.

** Weighted data.

### 3.2. Association between UHR and gynecological cancer history

In survey-weighted logistic regression analyses, each 1-percentage-point increase in UHR was associated with higher prevalence odds of self-reported gynecological cancer history in model 1 (OR 1.05, 95% CI: 1.02–1.07; *P* < .001), model 2 (OR 1.04, 95% CI: 1.01–1.06; *P* < .001), and model 3 (OR 1.03, 95% CI: 1.00–1.05; *P* = .03). The fully adjusted effect estimate was small, and the lower confidence bound rounded to 1.00. When UHR was analyzed by quartiles, none of the estimates for Q2, Q3, or Q4 differed significantly from that for Q1 in model 3, although the test for trend remained statistically significant. Thus, the quartile analyses did not support either a categorical threshold or a strong monotonic association. The continuous estimate should be interpreted as a weak association with prevalent cancer history rather than as evidence of increased cancer incidence or future risk (Table 3).

**Table 3:** The association between UHR and gynecological tumors by logistic regression analyses.

		Model 1		Model 2		Model 3	
		OR (95% CI)	*P*-value	OR (95% CI)	*P*-value	OR (95% CI)	*P*-value
UHR		1.05 (1.02–1.07)	**<.001**	1.04 (1.01–1.06)	**<.001**	1.03 (1.00–1.05)	**.03**
UHR (quartile)							
Q1	5.01 ± 0.90	Reference		Reference		Reference	
Q2	7.27 ± 0.59	1.00 (0.72–1.38)	1.00	0.98 (0.71–1.36)	.93	0.93 (0.67–1.30)	.68
Q3	9.58 ± 0.79	1.37 (1.01–1.86)	**<.05**	1.36 (1.00–1.84)	**<.05**	1.25 (0.91–1.72)	.17
Q4	14.55 ± 3.55	1.58 (1.18–2.12)	**.002**	1.48 (1.10–2.01)	**.009**	1.27 (0.92–1.76)	.15
*P* for trend			**<.001**		**.002**		**<.05**

Model 1: no covariates were adjusted. Model 2: adjusted for age, race. Model 3: adjusted for age, race, educational attainment, ratio of income to poverty level, alcohol, smoking, BMI, use of birth control pills, pregnancy status, marital status.

The bold values mean *P* < .05.

BMI = body mass index, CI = confidence interval, OR = odds ratio, UHR = uric acid-to-high-density lipoprotein cholesterol ratio.

### 3.3. Exploratory subgroup analyses

**Figure 2.**
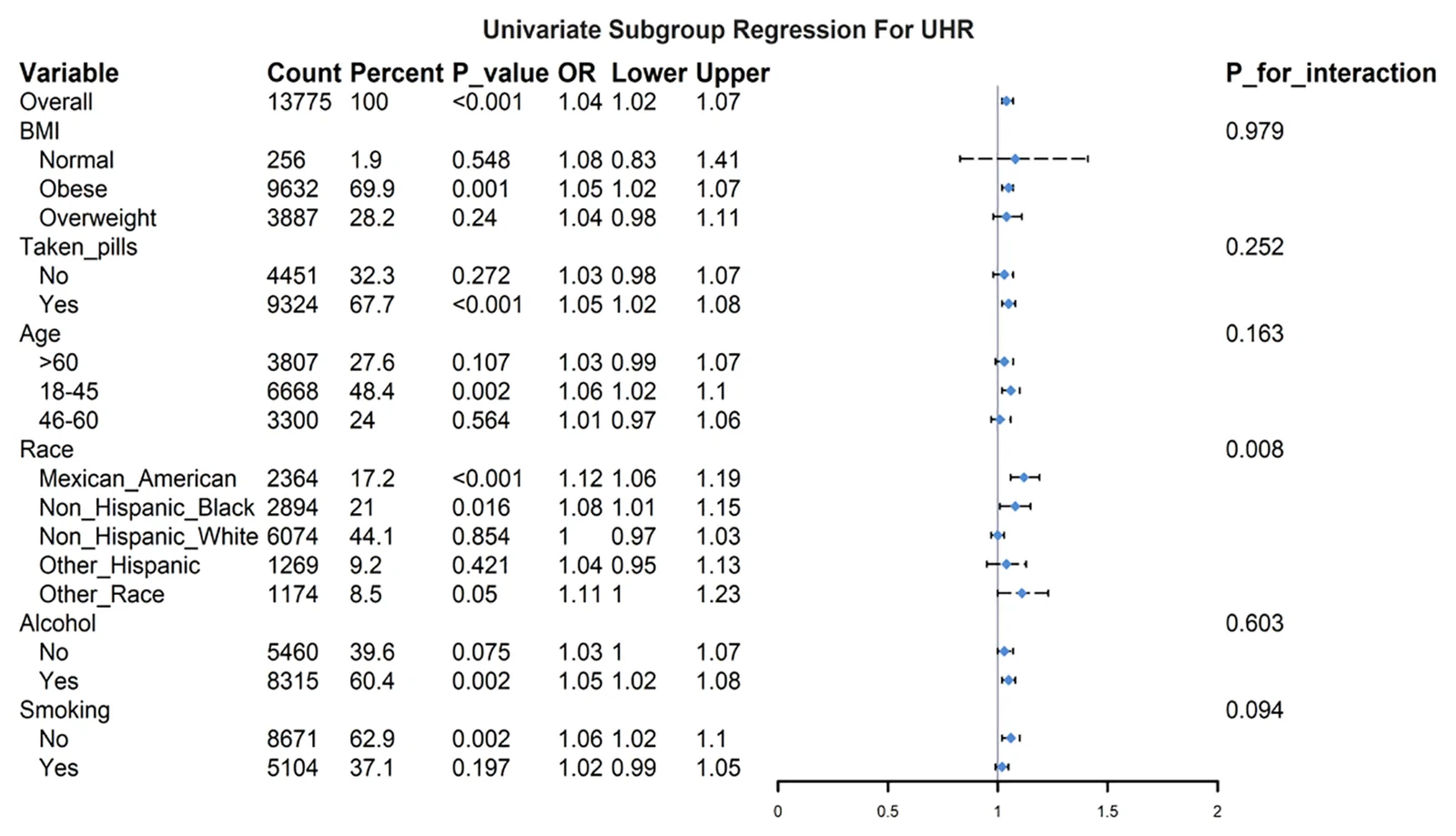
Exploratory association between UHR and self-reported gynecological cancer history across prespecified subgroups of age, BMI, smoking status, and oral contraceptive use. Estimates are based on model 3, excluding the stratification variable. *P* values for interaction are reported. No adjustment for multiple testing was applied; the findings are hypothesis-generating. BMI = body mass index, OR = odds ratio, UHR = uric acid-to-high-density lipoprotein cholesterol ratio.

Exploratory subgroup analyses evaluated the association between UHR and gynecological cancer history across categories of age, BMI, smoking status, and oral contraceptive use (Fig. 2). Some within-stratum estimates reached nominal statistical significance; for example, the association was observed among women aged 18 to 45 years (OR 1.06, 95% CI: 1.02–1.10; *P* = .002) and among oral contraceptive users (OR 1.05, 95% CI: 1.02–1.08; *P* < .001). However, within-stratum *P* values were not interpreted as evidence of effect modification. No interaction was considered robust given the limited number of outcome events and the multiple comparisons performed. Accordingly, these findings should be regarded as hypothesis-generating.

### 3.4. Dose–response and discrimination analyses

Survey-weighted RCS analyses did not detect a statistically significant departure from linearity for UHR (*P* for nonlinearity > .05). This finding indicates only that the available data did not provide clear evidence of nonlinearity; it does not establish a biological dose–response relationship. Analyses of sUA and HDL-C were exploratory and likewise cannot identify causal pathways.

**Figure 3.**
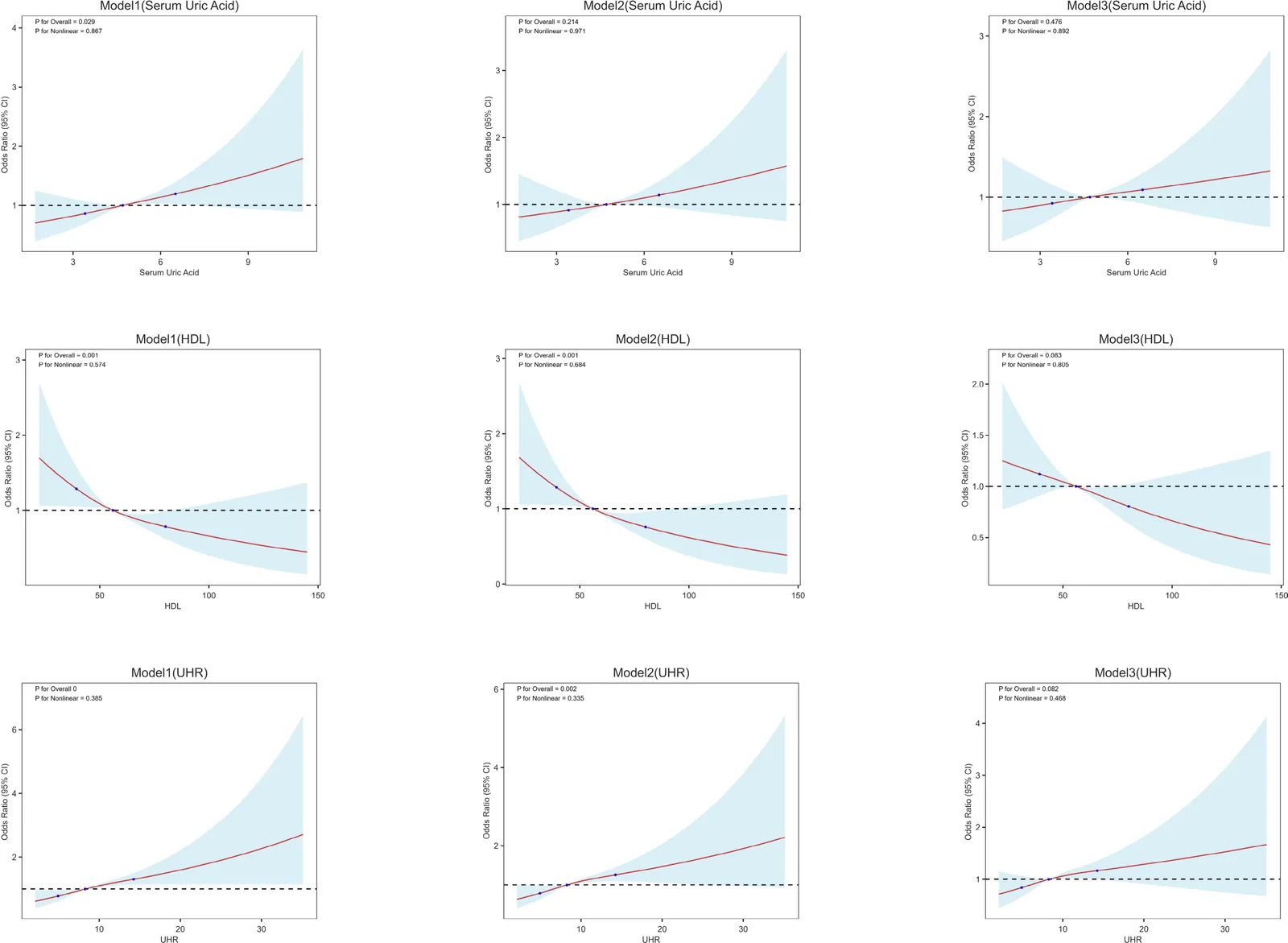
Survey-weighted restricted cubic spline associations between UHR and self-reported gynecological cancer history in models 1 to 3. The *P* value for nonlinearity should be reported. The absence of statistically significant nonlinearity does not establish a biological dose–response relationship. Model 1: unadjusted. Model 2: adjusted for age and race/ethnicity. Model 3: adjusted for age, race/ethnicity, educational attainment, poverty–income ratio, alcohol use, smoking status, BMI, oral contraceptive use, pregnancy history, and marital status. Odds ratios are prevalence odds ratios. Bold values indicate *P* < .05; however, interpretation should prioritize effect size and 95% confidence intervals. Odds ratios are prevalence odds ratios. Bold values indicate *P* < .05, but interpretation should prioritize effect size and 95% confidence intervals. BMI = body mass index, CI = confidence interval, HDL = high-density lipoprotein, UHR = uric acid-to-high-density lipoprotein cholesterol ratio.

**Figure 4.**
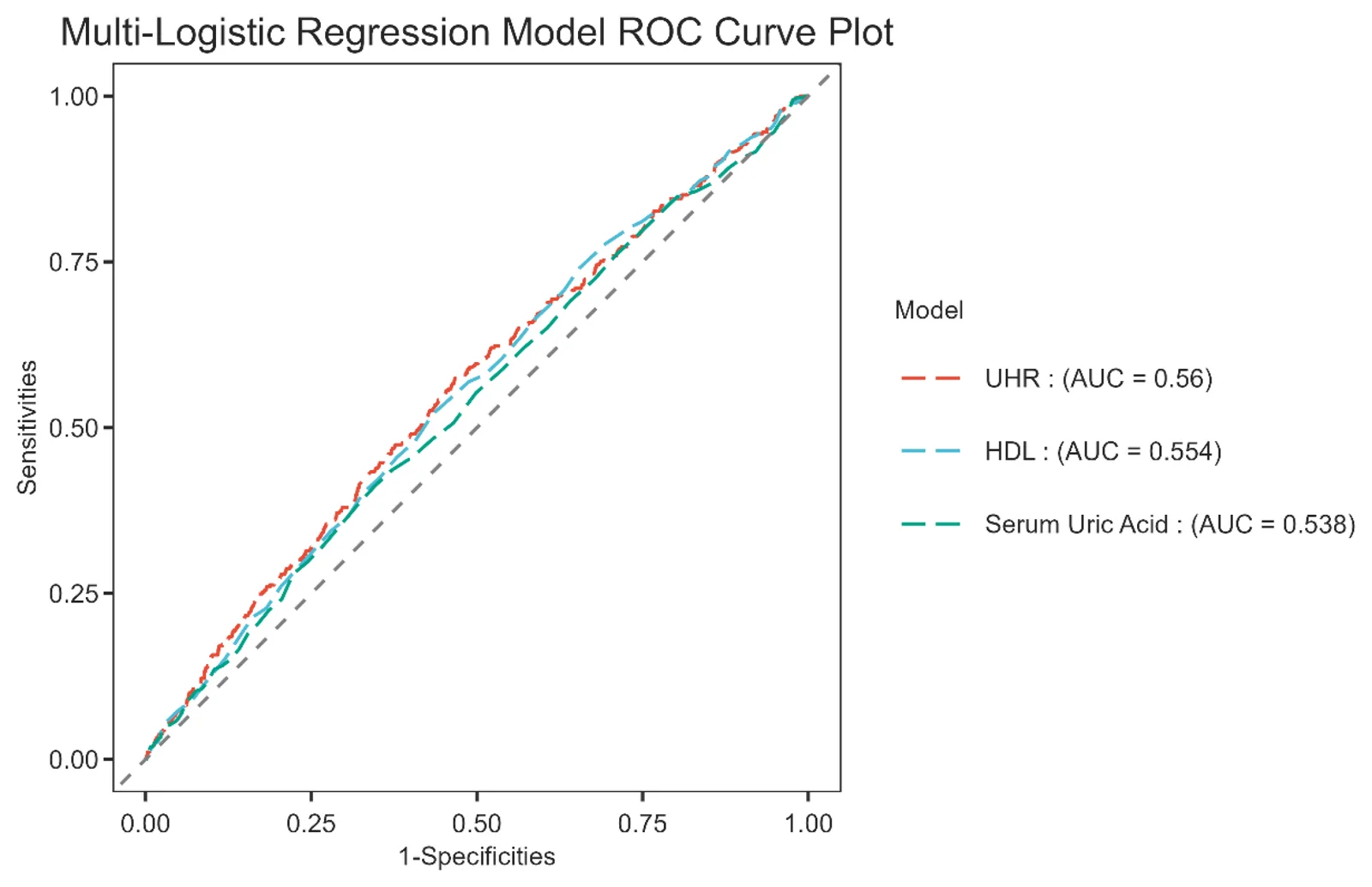
Exploratory ROC curves for UHR, sUA, and HDL-C. The observed AUCs (0.560, 0.538, and 0.554, respectively) indicate poor apparent discrimination and do not constitute validation of a prediction or screening model. AUC = area under the curve, HDL-C = high-density lipoprotein cholesterol, ROC = receiver operating characteristic, sUA = serum uric acid, UHR = uric acid-to-high-density lipoprotein cholesterol ratio.

ROC analysis yielded AUCs of 0.560 for UHR, 0.538 for sUA, and 0.554 for HDL-C. All values were close to 0.50, indicating poor apparent discrimination. The small numerical difference between UHR and its individual components was not evaluated for clinical significance, and neither internal nor external validation was performed. These findings, therefore, do not support characterizing UHR as a robust predictor or screening tool. The *E*-value for the fully adjusted continuous-UHR estimate was 1.20, and the *E*-value for the confidence limit closest to the null was 1.00, indicating that relatively weak unmeasured confounding could account for the observed association (Figs. 3 and 4).

## 4. Discussion

UHR has emerged as a composite marker of metabolic dysfunction because it integrates serum uric acid and HDL-C, 2 biomarkers with potentially complementary relationships to oxidative stress, inflammation, and cardiometabolic health.^[10]^ Interest in the relevance of UHR to female reproductive and gynecological conditions has increased. Recent NHANES-based studies reported positive associations between UHR and female infertility, particularly among younger women.^[11,12]^ UHR has also been associated with vascular complications in patients with type 2 diabetes, with stronger associations reported among older adults, women, and individuals with higher BMI.^[13]^ These patterns may partly reflect age- and adiposity-related increases in serum uric acid and reductions in HDL-C.^[14-16]^ Previous studies have also described dyslipidemia in patients with uterine and cervical cancers, including higher triglyceride, total cholesterol, and LDL-C levels and lower HDL-C levels.^[17-19]^ Endometrial cancer is additionally characterized by substantial alterations in lipid and intermediary metabolism, including changes in phosphocholine, insulin signaling, sterol regulatory element-binding protein 1, and sphingolipid pathways.^[18,20]^ Such metabolic abnormalities may contribute directly or indirectly to hyperuricemia.^[18,21]^ Collectively, these observations provide a plausible rationale for investigating UHR in relation to gynecological cancer, although they do not establish causality or clinical utility.

A growing body of research has linked UHR to several metabolic and chronic conditions, including chronic kidney disease, osteoarthritis, hypertension, nonalcoholic fatty liver disease, metabolic syndrome, obesity, and mortality among patients with diabetes.^[22-28]^ For example, Yazdi et al reported a higher mean UHR among individuals with metabolic syndrome than among those without the condition (14.76 ± 6.33% vs 10.00 ± 3.10%; *P* < .001), and a high UHR was associated with substantially greater odds of metabolic syndrome.^[26]^ Wang et al observed a positive association between UHR and obesity in older adults after adjustment for multiple potential confounders.^[27]^ Huang et al reported a U-shaped association of UHR with all-cause and cardiovascular mortality among patients with diabetes.^[28]^ Nevertheless, the biological mechanisms underlying UHR and the clinical implications of high or low values remain incompletely understood.

Metabolic disorders may influence cancer development and progression through multiple pathways,^[29]^ but evidence specifically linking UHR to cancer remains limited. Most existing studies have focused on serum uric acid or cholesterol metabolism separately.^[30-33]^ A meta-analysis by Yang et al reported a U-shaped association between serum uric acid and breast cancer risk, with the lowest estimated risk at approximately 4.5 mg/dL.^[30]^ Uric acid may exert both antioxidant and pro-oxidant effects: at physiological concentrations, it can scavenge reactive oxygen species, whereas elevated concentrations may promote oxidative stress, inflammation, endothelial dysfunction, insulin resistance, and proinflammatory signaling.^[34-39]^ Cholesterol metabolism is also closely involved in tumor biology.^[40,41]^ Malignant cells have increased cholesterol requirements, and cancer-associated metabolic changes may contribute to reduced circulating HDL-C levels.^[42]^ Observational studies have linked lower HDL-C levels to poorer prognosis and higher mortality among patients with cancer, whereas higher HDL-C levels have sometimes been associated with lower cancer risk.^[43-45]^ These findings support biological plausibility but remain vulnerable to confounding, reverse causation, and disease-related metabolic changes.

The ROC findings are particularly important for clinical interpretation. An AUC of 0.560 is only marginally above chance and is insufficient for individual-level classification. The present analysis did not evaluate calibration, net benefit, optimal thresholds, internal validation, external validation, or incremental predictive value beyond established risk factors. Therefore, UHR should not be proposed as a screening, early-detection, or risk-stratification marker on the basis of this study.

Several limitations should be emphasized. First, the cross-sectional design precludes temporal and causal inference and allows for reverse causation. Second, the outcome was based on a self-reported lifetime history of 3 heterogeneous cancers; the data did not distinguish active from previously treated disease, confirm diagnoses through cancer registries, or differentiate uterine cancer subtypes. Third, the complete-case analysis excluded 7818 women after the initial restriction to adult females, introducing the potential for selection bias; missing data were not imputed. Fourth, residual confounding is likely because menopausal status, hormone therapy, family history, HPV exposure or vaccination, diabetes, renal function, and inflammatory markers were not included. Fifth, the 369 outcome events limited statistical precision and the reliability of subgroup analyses. Sixth, multiple exploratory analyses were performed without adjustment for multiplicity. Finally, the generalizability of the findings to populations outside the United States and to specific gynecological cancer types is uncertain.

Future studies should use prospective cohorts or linked cancer registries, verify cancer type and diagnosis date, measure UHR before cancer diagnosis, prespecify confounders and subgroup hypotheses, and address missing data using appropriate methods. They should also evaluate whether UHR provides clinically meaningful incremental information beyond established risk factors. Any prediction model would require assessment of calibration and decision-curve utility, as well as rigorous internal and external validation before clinical application.

## 5. Conclusion

Among US women participating in NHANES 2003–2016, higher UHR was weakly associated with greater prevalence odds of a self-reported history of cervical, ovarian, or uterine cancer. The small effect size, nonsignificant fully adjusted quartile estimates, poor discriminatory performance, and limited robustness to unmeasured confounding do not support causal, screening, early detection, or risk prediction claims. These findings should be considered hypothesis-generating.

## Author contributions

**Conceptualization:** Zelong Li, Hui Xu, Sensen Shi.

**Data curation:** Zelong Li, Hui Xu, Sensen Shi.

**Formal analysis:** Zelong Li, Hui Xu, Sensen Shi.

**Investigation:** Zelong Li, Sensen Shi.

**Writing** – **original draft:** Sensen Shi.

**Writing** – **review & editing:** Sensen Shi.
